# Unsuccessful aortic fenestration for aortic dissection complicated with mesenteric malperfusion analyzed using computational fluid dynamics: a case report

**DOI:** 10.1186/s44215-025-00212-7

**Published:** 2025-06-17

**Authors:** Shoki Iwanaga, Naoyuki Kimura, Shuta Imada, Mutsumi Mizoguchi, Mamoru Arakawa, Hirohiko Akutsu, Koji Kawahito, Masanori Nakamura

**Affiliations:** 1 Department of Surgery, Division of Cardiovascular Surgery, Jichi Medical University, Yakushiji 3311-1, Shimotsuke, 329-0498 Japan; 2 Department of Electrical and Mechanical Engineering, Nagoya Institute of Technology, Gokiso, Showa, Nagoya, 466-8555 Japan

**Keywords:** Aortic dissection, Mesenteric malperfusion, Aortic fenestration, Computational fluid dynamics

## Abstract

**Background:**

We report a computational fluid dynamics (CFD)-based analysis of an unsuccessful open fenestration for aortic dissection with mesenteric malperfusion.

**Case presentation:**

A 75-year-old male was admitted for acute type B aortic dissection complicated by mesenteric malperfusion. He had a concomitant infrarenal abdominal aneurysm, prompting surgical infrarenal fenestration. Intraoperatively, the proximal intimal flap was resected near the renal arteries, and the aneurysm was replaced with a prosthetic graft. Despite the intervention, mesenteric malperfusion worsened, requiring additional endovascular aortic repair. CFD analysis revealed persistent false lumen flow and true lumen compression due to a large entry tear and residual proximal anastomotic stenosis.

**Conclusion:**

CFD analysis suggests that a large entry tear and residual stenosis from insufficient fenestration may result in inadequate false lumen depressurization.

**Supplementary Information:**

The online version contains supplementary material available at 10.1186/s44215-025-00212-7.

## Background

Aortic fenestration is a widely recognized treatment for organ malperfusion due to aortic dissection [1, 2]. Depressurizing the false lumen (FL), either by reducing blood flow into the FL or enhancing its drainage, is the primary therapeutic goal of both thoracic endovascular aortic repair (TEVAR) and aortic fenestration. Although endovascular therapy is the first-line treatment for organ malperfusion in acute type B aortic dissection (ATBAD), open surgical fenestration remains a viable alternative when endovascular therapy is unavailable. Here, we present a computational fluid dynamics (CFD)-based analysis of an unsuccessful open fenestration to explore the underlying mechanisms of failure.

## Case presentation

**Fig. 1:**
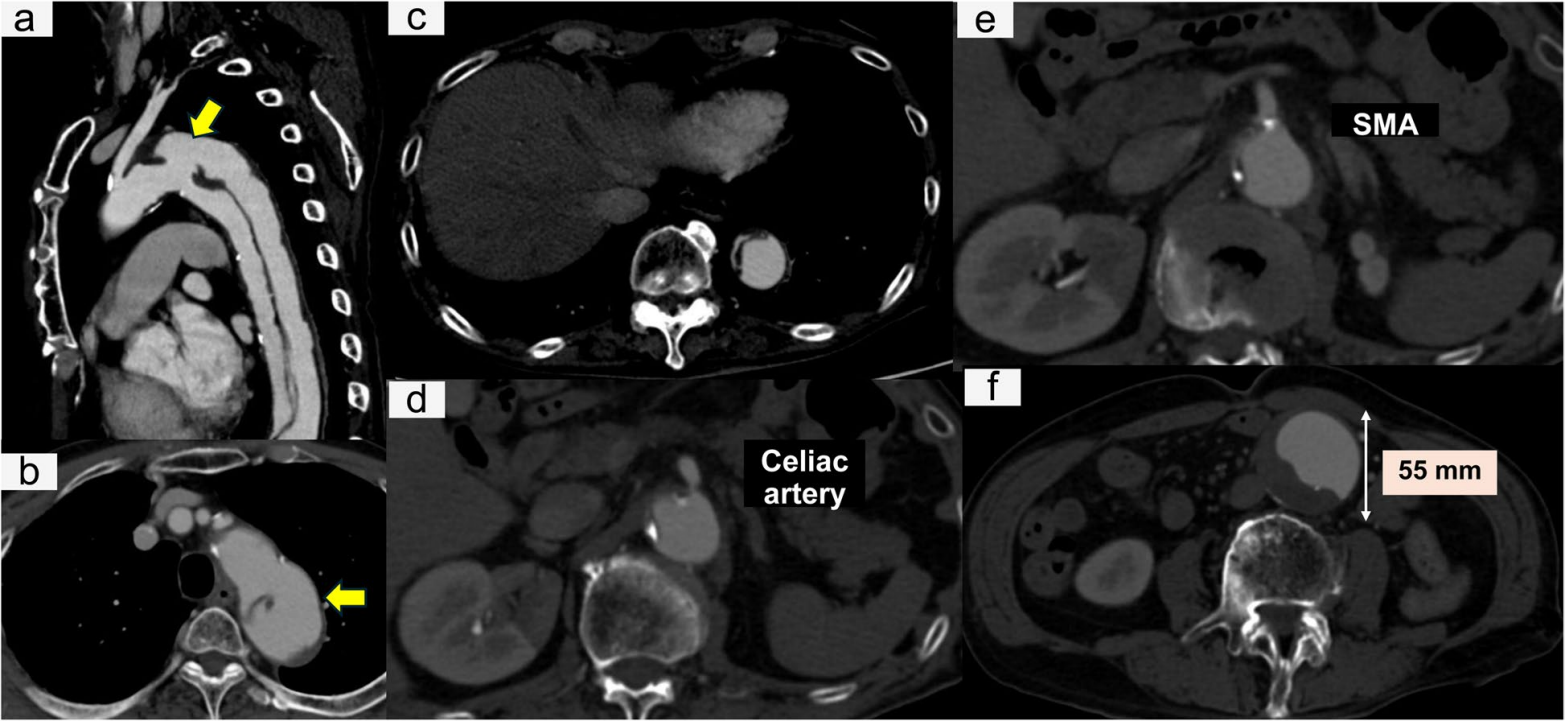
Preoperative computed tomography images. Sagittal (**a**) and axial (**b**) images show the entry tear (yellow arrow) in the descending aorta. Axial images reveal a severely compressed TL (**c**, **d**, **e**), the celiac artery originating from the FL (**d**), the SMA arising from the narrowed TL (**e**), and a 55-mm abdominal aortic aneurysm (**f**). TL, true lumen; FL, false lumen; SMA, superior mesenteric artery

A 75-year-old male was transferred to our hospital with sudden onset of abdominal pain. Computed tomography (CT) revealed a 21.7-mm entry tear in the proximal descending aorta, a patent FL extending just proximal to the abdominal aortic aneurysm (AAA), true lumen (TL) narrowing in the distal aorta, the celiac artery originating from the FL, the superior mesenteric artery arising from the narrowed TL, and a 55-mm infrarenal AAA (Fig. 1). The visceral vessels were not dissected. A diagnosis of ATBAD was made. Initial conservative management was chosen due to pain remission. However, on hospital day 2, the abdominal pain recurred with leg ischemia and an increase in serum lactate from 2 to 8 mmol/L. Dynamic obstruction exacerbation was suspected.

Surgical aortic fenestration was performed to depressurize the FL and resect the AAA, as an appropriately sized stent graft for TEVAR was not available in the emergency setting. After a median laparotomy, no signs of intestinal ischemia were seen. The abdominal aorta was clamped just below the renal arteries, and the bilateral common iliac arteries were clamped distally. The aneurysm was opened longitudinally, revealing the TL ending at the aneurysmal neck. The intimal flap was widely resected in a triangular fashion up to the aortic clamp. The proximal anastomosis was reinforced with Teflon felt, and a bifurcated prosthesis was implanted at the aneurysm site. The distal anastomosis was constructed in the bilateral common iliac arteries to create a single lumen. Intraoperative angiography was not performed to assess visceral perfusion. Postoperatively, leg malperfusion improved; however, on postoperative day (POD) 1, serum lactate rose to 10 mmol/L. CT showed persistent TL narrowing in the downstream aorta and signs of intestinal ischemia (Supplementary Fig. 1, Additional File 1). Exploratory laparotomy revealed 60 cm of necrotic ileum, which was resected. TEVAR was subsequently performed using a 26 × 80 mm Zenith dissection stent graft, a 31 × 200 mm conformable Gore TAG endoprosthesis, and a 36 × 164 mm Zenith dissection bare stent. Detailed procedural steps are described in Additional File 1. After TEVAR, serum lactate decreased, and oral intake resumed on POD 12. The patient was transferred for rehabilitation on POD 60. Postoperative CT confirmed TL expansion in the downstream aorta (Supplementary Fig. 2).

**Fig. 2:**
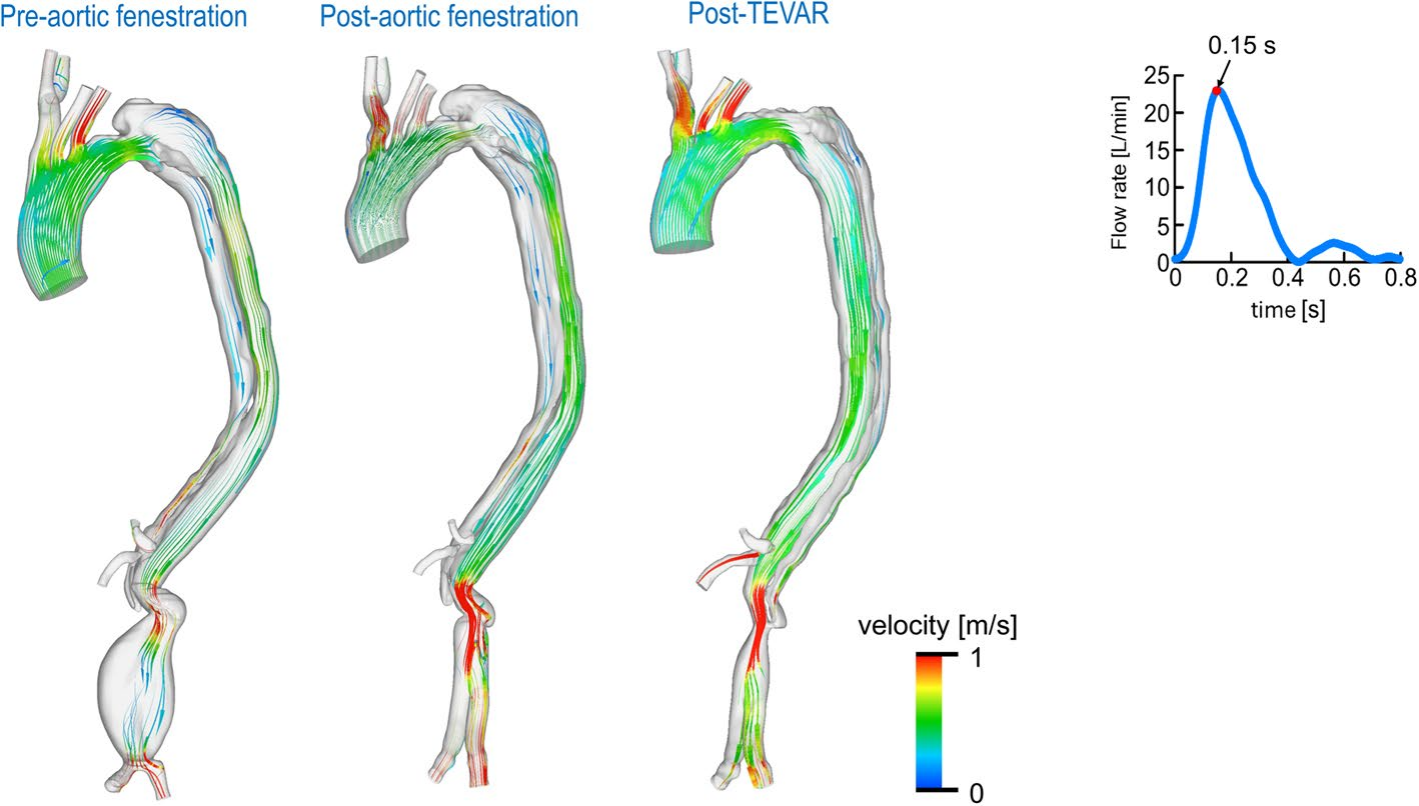
Streamline analysis of the aorta at mid-systole. TEVAR, thoracic endovascular aortic repair

**Fig. 3:**
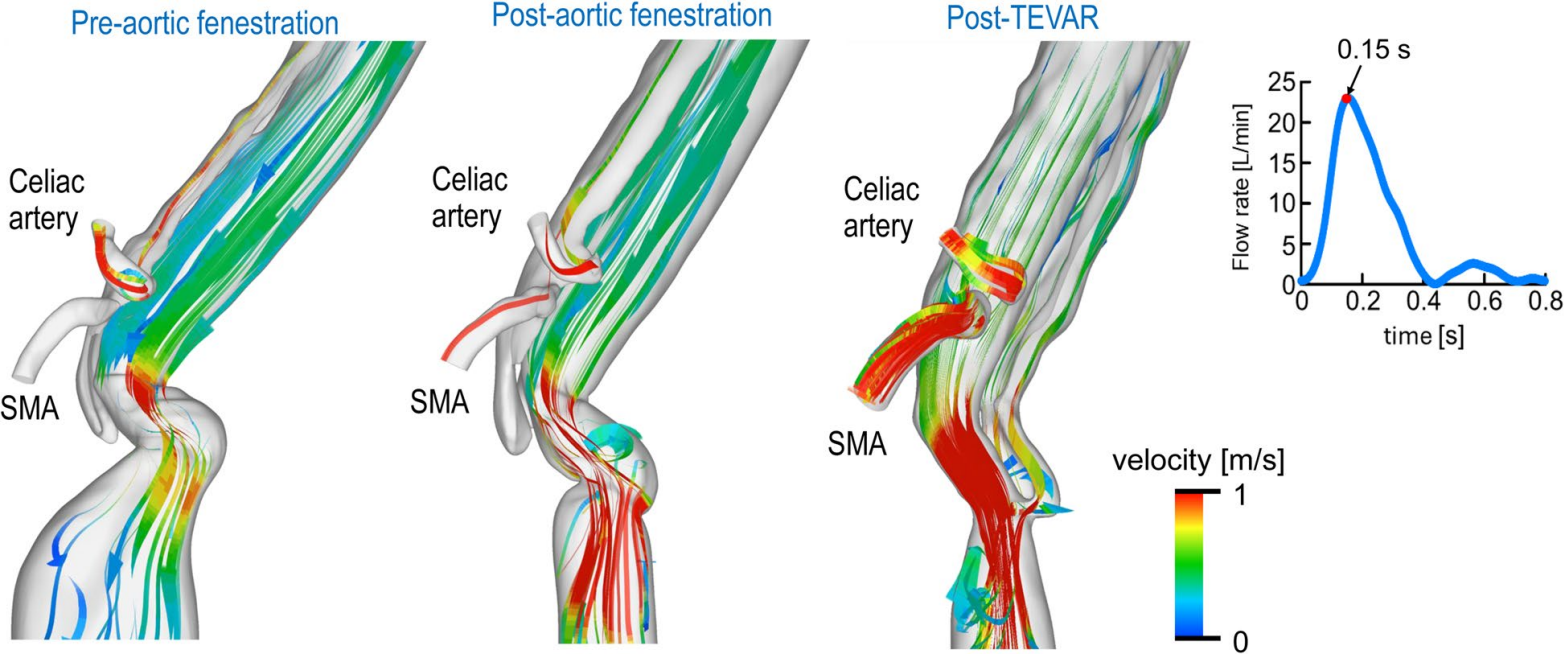
Streamline analysis of the visceral vessels at mid-systole. TEVAR, thoracic endovascular aortic repair

**Fig. 4:**
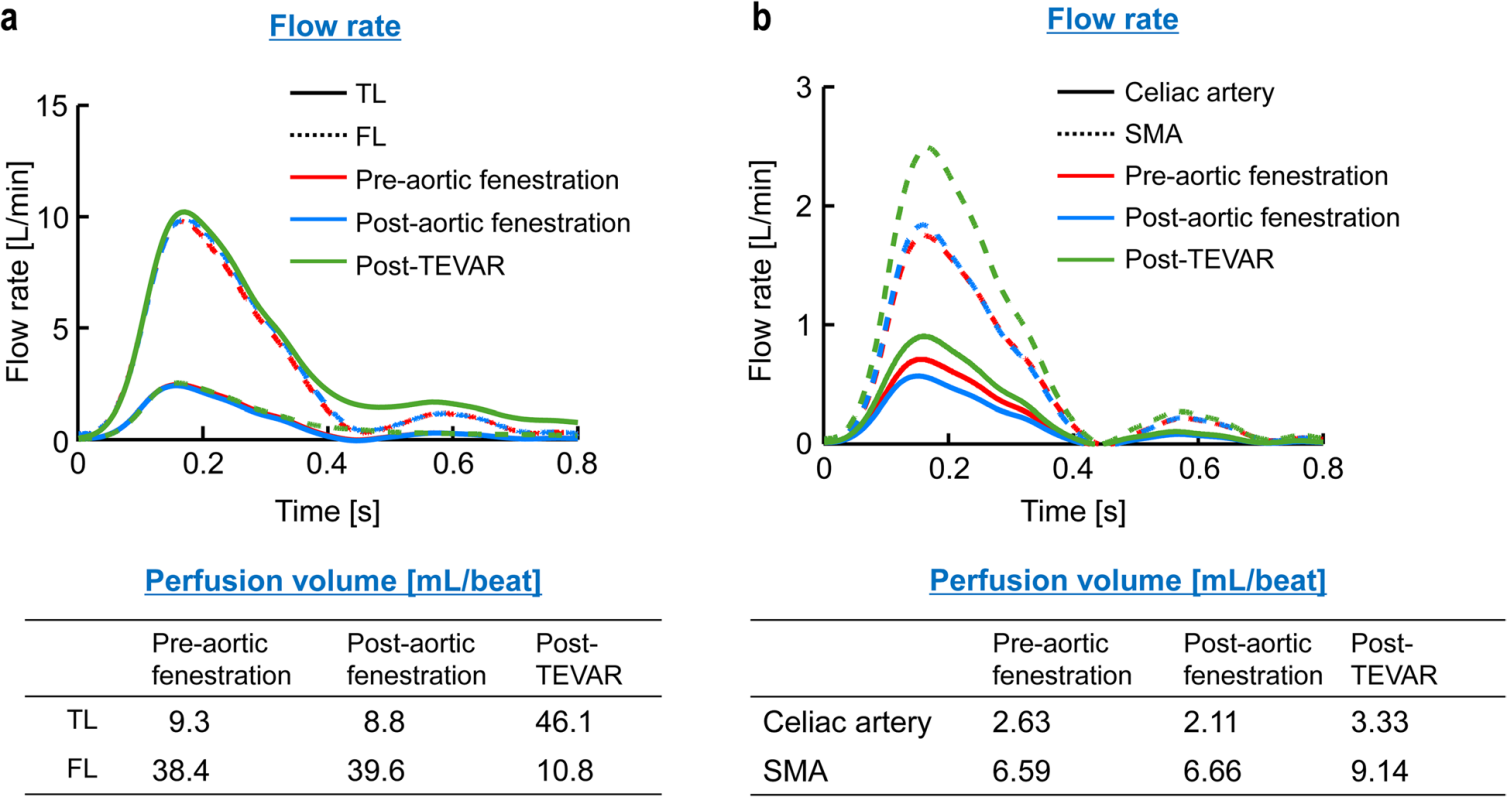
Line graph showing pre- and post-operative procedural flow rates (**a**) TL and FL of the descending aorta at the diaphragm level. (**b**) Visceral vessels. TL, true lumen; FL, false lumen; TEVAR, thoracic endovascular aortic repair

Details of the CFD analysis are provided in Additional File 1 and Supplementary Fig. 3. Vascular resistance (*R*) was calculated using a structured tree model [3–5]. Streamline visualizations of aortic and visceral vessel flow are shown in Figs. 2 and 3 and Videos 1 and 2 (Additional File 1). Preoperative imaging showed blood flow entering the FL through a large entry tear (Fig. 2, Video 1). Post-fenestration imaging showed persistent high FL flow with inadequate TL flow and increased flow velocity in the abdominal aorta near the proximal anastomosis, indicating residual stenosis (Fig. 2, Video 1). After TEVAR, antegrade FL flow disappeared, and TL flow markedly improved (Fig. 2, Video 1). Consistent with this, TL expansion after TEVAR increased blood flow to the visceral vessels during systole (Fig. 3, Video 2). Quantitative data are shown in Fig. 4. Post-fenestration, TL flow rate at the diaphragm did not significantly increase. After TEVAR, TL flow rate and perfusion volume improved significantly (Fig. 4a). Similarly, visceral vessel flow rate and perfusion volume also increased (Fig. 4b).

## Discussion

Additional aortic interventions following fenestration are rare. In a cohort of 182 patients with ATBAD treated with endovascular fenestration or stenting, only 9 (4.9%) required further aortic interventions, including TEVAR or aortic repair, due to impending rupture or persistent symptoms [1].

Our CFD analysis identified high FL flow through a large entry tear, along with significant residual stenosis near the proximal anastomosis following fenestration. During fenestration, the intimal flap was resected only up to the level of the aortic clamp below the renal arteries. This limited resection, combined with the large entry tear, may have resulted in inadequate FL decompression, contributing to the failure of the fenestration. Moreover, the absence of intraoperative angiography may have delayed recognition of ongoing ischemia and the need for timely TEVAR.

While ATBAD with limb malperfusion following endovascular AAA repair has been reported [6], to our knowledge, ATBAD with mesenteric malperfusion in an untreated AAA has not. In such cases, when the risk of AAA rupture is low, management should prioritize resolving mesenteric malperfusion. The optimal approach depends on factors such as entry tear location and size, and the type of malperfusion (dynamic, static, or mixed). TEVAR is the first-line treatment for dynamic or mixed-type obstruction when technically feasible and rupture risk is low. For isolated static obstruction, endovascular or surgical revascularization should be considered. The timing and method of AAA repair—open vs. endovascular, staged vs. concomitant—should be based on aneurysm size and overall patient risk. Another option for mesenteric malperfusion is suprarenal aortic fenestration. In this case, suprarenal clamping and a more extensive resection of the intimal flap, closer to the origin of the visceral branches, might have better alleviated FL pressurization.

## Conclusion

The CFD analysis indicated that a large entry tear combined with insufficient abdominal fenestration may have led to inadequate FL depressurization.

## Supplementary Information

Additional file 1: Supplementary Fig. 1. CT images at 1 day following aortic fenestration revealing the unrelieved TL narrowing in the (a) descending thoracic aorta and (b) abdominal aorta. Computed tomography image also revealing (c) poor contrast-enhanced intestinal wall with pneumatosis intestinalis. CT, computed tomography; TL, true lumen. Supplementary Fig. 2. CT images at 6 days following TEVAR revealing an expanded TL in the (a) descending thoracic aorta and (b) abdominal aorta. CT, computed tomography; TEVAR, thoracic endovascular aortic repair; TL, true lumen. Supplementary Fig. 3. Procedural steps for patient-specific CFD simulation of blood flow. CFD, computational fluid dynamics; TEVAR, thoracic endovascular aortic repair. Video legends. Video 1. Streamline analysis of the aorta in a patient with acute type B aortic dissection complicated by mesenteric malperfusion. left: pre-aortic fenestration, middle: post-aortic fenestration, right: post-TEVAR. TEVAR, thoracic endovascular aortic repair. Video 2. Streamline analysis of the visceral vessels in a patient with acute type B aortic dissection complicated by mesenteric malperfusion. left: pre-aortic fenestration, middle: post-aortic fenestration, right: post-TEVAR. TEVAR, thoracic endovascular aortic repair; SMA, superior mesenteric artery.

## Data Availability

Not applicable.

## Abbreviations

FL: False lumen
TEVAR: Thoracic endovascular aortic repair
ATBAD: Acute type B aortic dissection
CFD: Computational fluid dynamics
CT: Computed tomography
AAA: Abdominal aortic aneurysm
TL: True lumen
POD: Postoperative day
