# Supplementary material for: Unsuccessful aortic fenestration for aortic dissection complicated with mesenteric malperfusion analyzed using computational fluid dynamics: a case report
Source: Gen Thorac Cardiovasc Surg Cases. 2025 Jun 17;4:28. doi: 10.1186/s44215-025-00212-7 (PMC12175397; doi:10.1186/s44215-025-00212-7)
Supplement: Supplementary file 1 — Additional file 1: Supplementary Fig. 1. CT images at 1 day following aortic fenestration revealing the unrelieved TL narrowing in the (a) descending thoracic aorta and (b) abdominal aorta. Computed tomography image also revealing (c) poor contrast-enhanced intestinal wall with pneumatosis intestinalis. CT, computed tomography; TL, true lumen. Supplementary Fig. 2. CT images at 6 days following TEVAR revealing an expanded TL in the (a) descending thoracic aorta and (b) abdominal aorta. CT, computed tomography; TEVAR, thoracic endovascular aortic repair; TL, true lumen. Supplementary Fig. 3. Procedural steps for patient-specific CFD simulation of blood flow. CFD, computational fluid dynamics; TEVAR, thoracic endovascular aortic repair. Video legends. Video 1. Streamline analysis of the aorta in a patient with acute type B aortic dissection complicated by mesenteric malperfusion. left: pre-aortic fenestration, middle: post-aortic fenestration, right: post-TEVAR. TEVAR, thoracic endovascular aortic repair. Video 2. Streamline analysis of the visceral vessels in a patient with acute type B aortic dissection complicated by mesenteric malperfusion. left: pre-aortic fenestration, middle: post-aortic fenestration, right: post-TEVAR. TEVAR, thoracic endovascular aortic repair; SMA, superior mesenteric artery. [file 44215_2025_212_MOESM1_ESM.zip › Additional_fileR2.docx]

**Additional File 1**

**Operative procedure regarding TEVAR**

Thoracic endovascular aortic repair (TEVAR) was performed to close the primary entry tear located at the proximal descending thoracic aorta and expand the true lumen (TL). Under general anesthesia, a 9-Fr sheath and guidewire were initially inserted via the right femoral artery. However, owing to an extremely stenotic intimal flap, the guidewire could not pass through the proximal anastomosis of the abdominal aortic replacement. Consequently, the guidewire was inserted via the left brachial artery to establish a pull-through wire in the TL, which was confirmed using intravascular ultrasonography, extending to the right femoral artery. Subsequently, a stiff guidewire was advanced into the ascending aorta via the right femoral artery.

The size of the TL was measured using computed tomography (CT), revealing an average proximal diameter of 33 mm and a distal longitudinal diameter of 26 mm. Initially, a Zenith dissection stent graft (diameter, 26 mm; length, 80 mm; Cook Medical, Bloomington, IN, USA) was deployed at the straight segment of the descending thoracic aorta. Subsequently, a conformable Gore TAG thoracic endoprosthesis (diameter, 31 mm; length, 200 mm; W. L. Gore and Associates, Flagstaff, AZ, USA) was placed to seal the primary entry tear. Finally, a Zenith dissection bare stent (diameter, 36 mm; length, 164 mm; Cook Medical, Bloomington, IN, USA) was deployed distal to the endoprosthesis and proximal to the bifurcated prosthesis implanted in the abdominal aorta. Although a small type Ia endoleak was observed, no additional stent was implanted, since the purpose of TEVAR was true lumen expansion. During the TEVAR, improvement of visceral perfusion was confirmed by intraoperative angiography.

**Computer simulation of the blood flow**

Computed tomography angiography (CTA) was preoperatively and postoperatively performed using a SOMATOM Definition Flash scanner (Siemens Healthcare, Munich, Germany). Scanning was performed at a 1-mm slice thickness. CT covered the entire aorta, including the three main branches arising from the aortic arch and the origin of the common iliac arteries.

The luminal geometries of the aorta, celiac artery, and superior mesenteric artery (SMA) were extracted from the preoperative and postoperative CTA, which had a spatial resolution of 0.877 × 0.877 × 0.5 mm and 0.782 × 0.782 × 0.5 mm, respectively. The geometries were reconstructed using the three-dimensional (3D) Slicer 4.10.2 open-source imaging software. Well-experienced cardiac surgeons confirmed the constructed geometries. A two-layer surface prism mesh was constructed, and a polyhedral mesh was constructed for the internal region. The total number of mesh elements was approximately 860,000.

Pulsatile blood flow in the aorta was simulated using a commercially available computational fluid dynamics program (scFLOW v2021, MSC Software Japan, Tokyo, Japan). Multiscale blood flow simulation was achieved by coupling the Navier–Stokes equations in a 3D domain to the peripheral blood flow models in a 0D domain (Supplementary Fig. 3). Peripheral blood flow modeling was based on the hydraulic–electric analog, wherein vascular resistance, pressure difference, and mass flow are represented by the electrical resistance, potential difference, and electrical current, respectively. The vascular resistance *R* was determined on the basis of a structured tree model [5-7]. The pressure at the downstream end of the 0D model was taken as the capillary pressure, *P_t_* = 30 mmHg [6]. A physiological flow rate with a heart rate of 75 beats/min and a stroke volume of 83.0 mL/beat at the aortic root was assigned as the inlet boundary condition. The wall was assumed to be rigid, and a no-slip boundary condition was applied. Blood was assumed to be an incompressible Newtonian fluid, with non-Newtonian behavior being negligible in large arteries. The density and dynamic viscosity were set to 1.05 × 10^3^ kg/m^3^ and 4.0 × 10^−3^ Pa/s, respectively.

Hemodynamic changes in the flow pattern affected by TEVAR were investigated using streamline analysis. Flow rate (L/min) and perfusion volume/cardiac cycle were calculated at (1) both the TL and false lumen of the descending thoracic aorta at the diaphragm, (2) celiac artery, and (3) SMA (Supplementary Fig. 3).

**Supplementary Fig. 1**

**
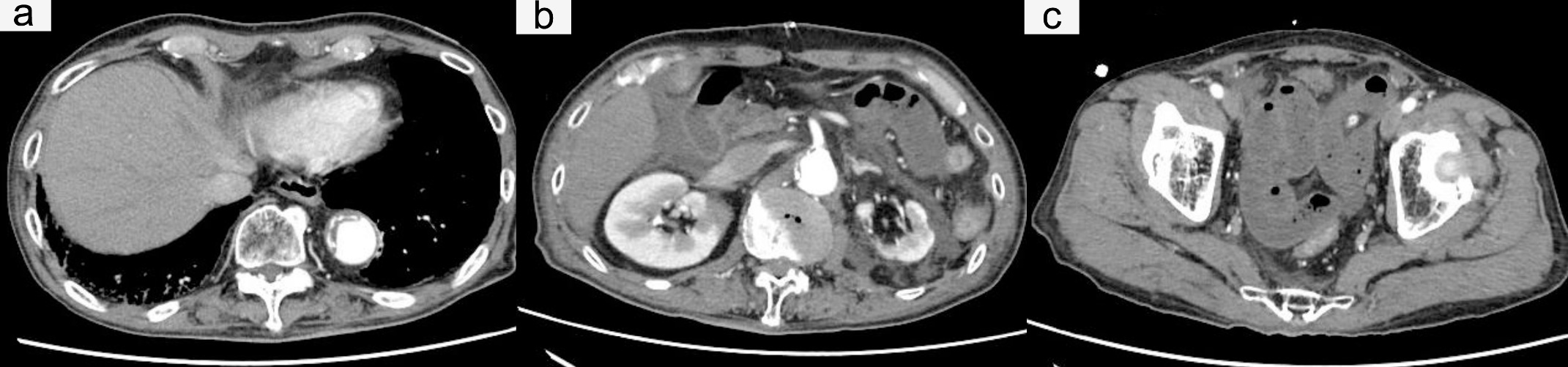
**

CT images at 1 day following aortic fenestration revealing the unrelieved TL narrowing in the (a) descending thoracic aorta and (b) abdominal aorta. Computed tomography image also revealing (c) poor contrast-enhanced intestinal wall with pneumatosis intestinalis. CT, computed tomography; TL, true lumen

**Supplementary Fig. 2**

**
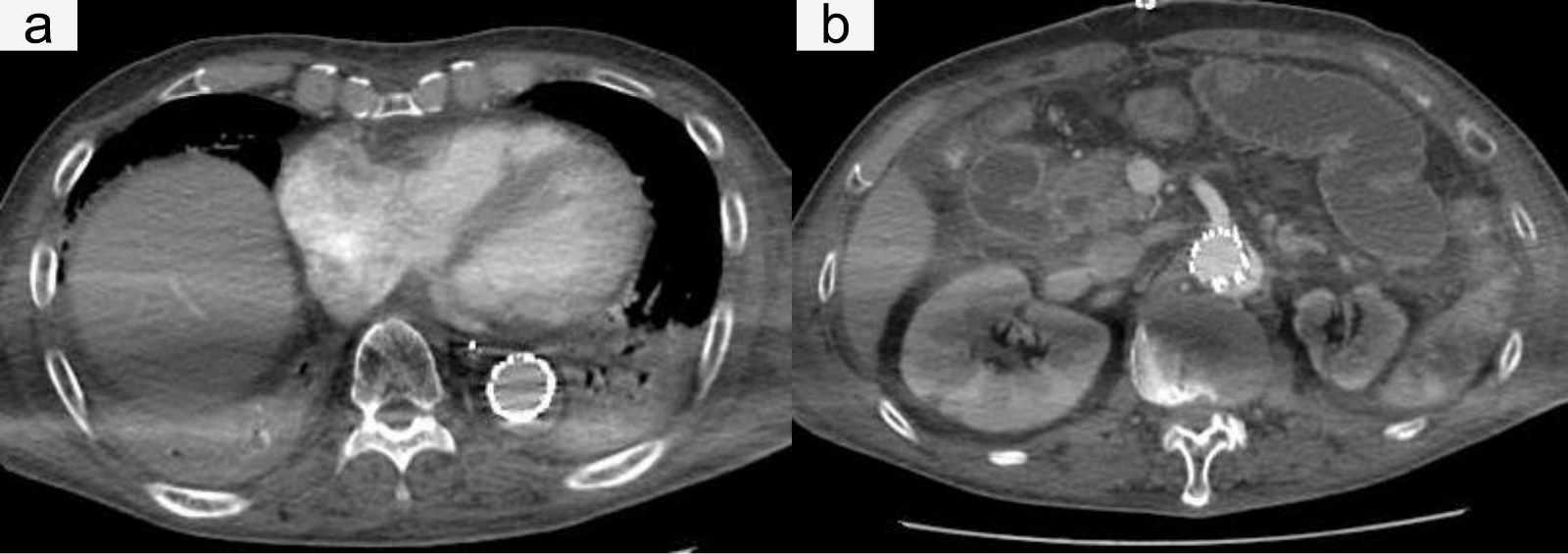
**

CT images at 6 days following TEVAR revealing an expanded TL in the (a) descending thoracic aorta and (b) abdominal aorta. CT, computed tomography; TEVAR, thoracic endovascular aortic repair; TL, true lumen

**Supplementary Fig. 3**

**
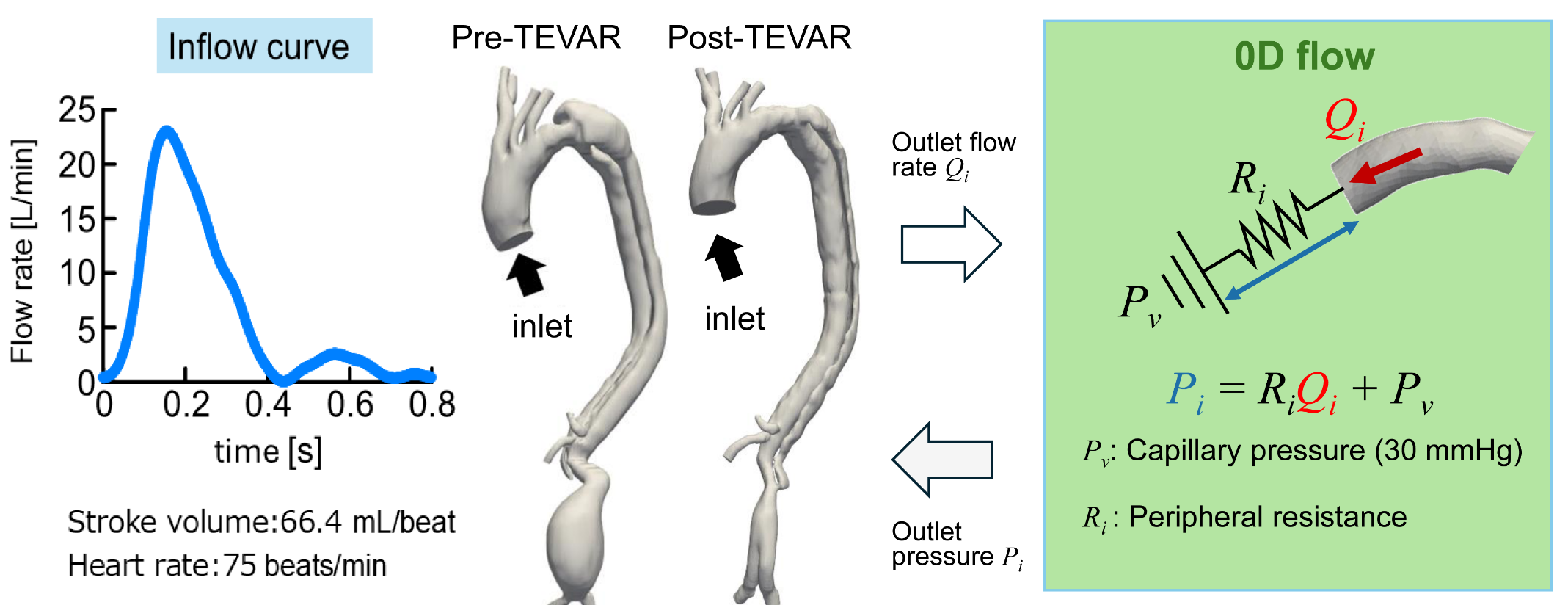
**

Procedural steps for patient-specific CFD simulation of blood flow. CFD, computational fluid dynamics; TEVAR, thoracic endovascular aortic repair

**Video legends**

**Video 1**

Streamline analysis of the aorta in a patient with acute type B aortic dissection complicated by mesenteric malperfusion. left: pre-aortic fenestration, middle: post-aortic fenestration, right: post-TEVAR. TEVAR, thoracic endovascular aortic repair

**Video 2**

Streamline analysis of the visceral vessels in a patient with acute type B aortic dissection complicated by mesenteric malperfusion. left: pre-aortic fenestration, middle: post-aortic fenestration, right: post-TEVAR. TEVAR, thoracic endovascular aortic repair; SMA, superior mesenteric artery
